# Night-Time Decibel Hell: Mapping Noise Exposure Zones and Individual Annoyance Ratings in an Urban Environment in Ghana

**DOI:** 10.1155/2014/892105

**Published:** 2014-07-17

**Authors:** Rachel N. Zakpala, Frederick Ato Armah, Brigid M. Sackey, Opoku Pabi

**Affiliations:** ^1^Institute for Environment and Sanitation Studies, University of Ghana, Legon, Accra, Ghana; ^2^Department of Environmental Sciences, University of Cape Coast, Cape Coast, Ghana; ^3^Centre for Social Policy Studies, University of Ghana, Legon, Accra, Ghana

## Abstract

Although accumulating evidence over the past thirty years indicates that noise is an environmental stressor in residential settings, much of the data emanated from studies in high-intensity, noise impact zones around airports or major roads. Little is known about religious noise, especially at night, which is increasingly a growing concern for both the general public and policy-makers in sub-Saharan Africa. Using geographical information systems (GIS), this study measured and mapped exposure to religious noise in a rapidly urbanising municipality in Ghana. Quantitative noise risk assessment was used to evaluate the risk of religious noise-induced hearing loss to residents in the exposed neighbourhoods. The results show that all neighbourhoods where churches were situated had at least one location with significant risk of noise-induced hearing loss. However, there was no statistically significant relationship between neighbourhoods where religious noise exposure was the highest and where noise annoyance was the highest. The magnitude of the noise values for night-time exposure is remarkable particularly given that excessive night-time noise exposure has the greatest detrimental effect on public health. There is the need to focus on vulnerable groups, sensitive hours of the night, and possible confounding with air pollution in order to wholly address this potential hazard.

## 1. Introduction

The proliferation of environmental noise is a defining characteristic of the 21st century [1]. Despite attempts to regulate it, noise pollution has become an unfortunate fact of life worldwide. Analogous to second-hand smoke, second-hand noise is an unwanted airborne pollutant produced by others [1]. In urban environments, air and road traffic have been the major sources of environmental noise. Assessment of exposure to noise requires consideration of many factors, including measured exposure or calculated/predicted exposure, choice of noise indicator, population distribution, time-activity patterns of the exposed population, and combined exposures to multiple sources of noise [2]. Social and behavioural effects of noise exposure are complex, subtle, and indirect. These effects include changes in everyday behaviour, changes in social behaviour, changes in social indicators, and changes in mood [1].

Sleep is an essential behaviour that provides physical and mental restoration and sleep disturbances are considered deleterious to mood, performance, and health [3]. Religious noise has become a major source of sleep disturbances and this situation will worsen with increasing church and mosque density within the forthcoming years and here more during the night than during the day. Night-time environmental noise exposure is an issue of enduring importance to urban planners, policy-makers, and researchers.

In 2009, the WHO published the night noise guidelines for Europe. This publication presented new evidence of the health damage of night-time noise exposure and recommended threshold values that if breached at night, would threaten health. Night-time noise disturbance during sleep is regarded as one of the most important aspects of environmental noise exposure with possible effects on health [4–6]. Exposure to night-time noise can also produce a number of secondary effects (i.e., those that can be measured the day after the individual is exposed to night-time noise) including psychological and physiological symptoms as well as reduced performance in adults [7].

The noise sensitivity of the sleeper depends on a plethora of factors some of which are noise dependent, such as the type of noise (e.g., continuous, intermittent, and impulsive), noise intensity, noise frequency, noise spectrum, noise interval (e.g., duration, regularity, expected), noise signification, and the difference between the background noise level and the maximum amplitude of the occurring noise stimulus [8]. Other factors are related to the sleeper, such as age, sex, personality characteristics, and self-estimated sensitivity to noise [8]. Noise-dependent and sleeper-dependent factors thus constitute a complex set of factors that jointly shape the responses of individuals and the population to night-time noise exposure.  Table 1 shows the relationship of night-time noise exposure and adverse health outcomes.

Hitherto, attention to night-time noise exposure has focused on air and road vehicular traffic in urban environments. An often neglected source of night-time noise exposure is religious noise which is fast becoming a defining characteristic of developing countries especially in sub-Saharan Africa. Religious noise adds to other sources of noise. For instance, if an individual works in a noise intensive environment during the daytime, the night-time exposures will cumulate whatever dose has already accrued. As a measure of the growing concern in relation to this issue, problems with religious noise have recently been rated at the highest level of environmental concern in some west African countries including Ghana. According to Armah et al. [9], religious noise especially in urban residential settings is a fast growing issue that requires research and policy attention. Religious noise exposure at night is pervasive in developing countries but has received little attention in the burgeoning literature on noise pollution in urban environments.

Noise characterises Christian, Muslim, and African traditional religious activities in Ghana [10–13]. This should be seen in the context of the balance between religious freedom and the public interest in environmental protection [14]. In this regard, public debate on religious noise in Ghana revolves around the following issues: (1) whether the right to practice religion is absolute and is not subject to limitations of “public order, morality, or health”; (2) whether, in a civilised society, religious activities disturbing the peace of others can be justified (noting that no religion prescribes or preaches that prayers must be performed through voice amplifiers or by beating drums) and whether there is a religious right to breach the permissible limits of the Environmental Protection Agency Act; and (3) even if the noise pollution in certain cities may already exceed those limits, whether it is sufficient ground for permitting others to increase the noise. Night-time Christian religious services are of three types: normal, half-night, and all-night, each of which typically lasts for up to 2.5, 5, and 8 hours, respectively. The exposure duration of the latter is thus equivalent to occupational noise although the exposure frequency of night-time Christian religious services may be different from occupational noise. Typically, night services do not exceed five days per week. However, it is also not uncommon to have night services every day of the week during special occasions.

**Figure 1:**
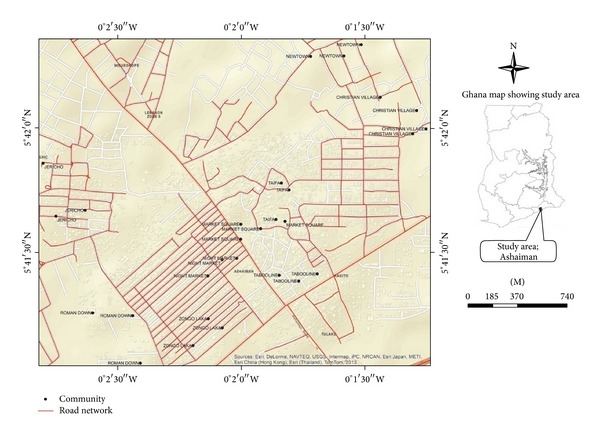
Map of Ashaiman showing the selected communities.

Our understanding of religious noise exposure is rather nascent. To our knowledge, no study has till date considered spatial distribution of night-time religious noise exposure in urban residential environments. This study therefore sought to measure the levels of religious noise produced in the Ashaiman Municipality, a rapidly growing urban environment in Ghana, to construct a night-time noise map indicating the distribution of exposure in residential neighbourhoods of the Ashaiman Municipality, and to assess the risk of religious noise-induced hearing loss to residents in the exposed neighbourhoods. Given the rapid increase in density of buildings for religious activities in Ghana in recent years, it is hypothesised that exposure to religious noise in the study area is considerably above the Ghana Environmental Protection Agency limits of 48 dB(A) *L*_eq_ for night-time exposure. This limit is almost identical to the World Health Organisation limit of 45 dB(A) *L*_eq_ for night-time exposure [15]. These limits are regarded as the levels above which exposure to noise is detrimental to human health. In order to investigate the hypothesis, the measurements of noise levels were taken at selected locations in the study area. It is anticipated that this study will inform environmental noise pollution policy in Ghana and the west African subregion Figure 1 shows the communities in which the study was undertaken.

## 2. Materials and Method

### 2.1. Study Area

The Ashaiman Municipality is one of the ten (10) districts in the Greater Accra Region of Ghana. Its capital is Ashaiman and was created in 2008. Ashaiman has coordinates 5° 42′ north and 0° 01′ west. It is located about four kilometers (4 km) to the north of Tema and about thirty kilometers (30 km) from Accra, the capital city of Ghana. Ashaiman shares boundaries to the north and east with Katamanso traditional area, to the south with the Tema Township, and to the west with Adjei Kojo, all within Tema Metropolitan Assembly. It has many communities some of which are planned and the rest unplanned. Specifically, this study focuses on the following residential communities in the Ashaiman Municipality: Christian Village, Zongo Laka, Roman Down, Market Square, Taifa, Taboo Line, New Town, Jericho, and Night Market. These communities were selected based on the density of the religious noise sources. According to the 2010 census, Ashaiman has 240,000 inhabitants of which 80% are Christians with majority of them (37.4%) belonging to the Pentecostal/Charismatic denomination. Nine percent (9%) Muslims and 11% either belong to the traditional African religions or have no religion [16]. Most of the inhabitants are into trading activities. Some inhabitants are engaged in the agriculture sector (crop farming, livestock rearing, and fishing).

### 2.2. Selection of Communities, Churches and Mosques, and Respondents

The selection of communities was done using a stratified random sampling technique. The communities were stratified into the settlement patterns of planned and unplanned areas. The communities were then selected from the two groups to adequately represent Ashaiman. Three communities were selected from the planned settlements, namely, Christian Village, Roman Down, and New Town. Zongo Laka, Market Square, Taifa, Taboo Line, Jericho, and Night Market were the other communities sampled from the unplanned areas. Selection of houses of respondents was based on the various distances from the noise sources, namely, 50 m, 100 m, 150 m, and 200 m. This was based on the fact that intensity varies from the source outwards. The heads of each household were interviewed. However, in the absence of the head of the household, any person available in the selected houses capable of providing authentic answers to the questions was interviewed. Data collection was conducted using a face-to-face personal interview on randomly selected participants aged between the ages of 18 and 79 years. To be eligible for study, all interviewees were at least 18 years old and had continuously lived in the neighbourhood for a minimum of 3 years. The personal interview collected self-reported information such as basic demographics, perception of noise, health, and quality of life indicators. Responses to noise were also assessed through objective measures of health such as sleep disturbance.

The churches and mosques were selected by purposive sampling technique. Selection of churches was done based on two criteria. First, the category of church, that is, either Orthodox churches, Pentecostal churches, or Charismatic church, and secondly the physical structure, that is, only churches operating in permanent physical structures, were considered. This was to ensure that the churches would always be there and would not relocate in the course of the study. The number of churches sampled was however based on those that could be assessed effectively and efficiently and within the time and material and financial resource limits of the study. The study population included individuals of both sexes under 18, 18–60, and those above 60 years. The participants who were within 18–60 years and those above 60 years were either heads of the families or not and were capable of providing authentic information for the study. In all 220 respondents were interviewed (Table 2).

### 2.3. Sound-Level Measurement

A precision-grade sound-level meter RION NL-22 (Higashimotomachi, Tokyo, Japan) was used to measure the noise levels generated at the churches and mosques and the various distances of 50 m, 100 m, 150 m, and 200 m away from the churches and mosques. The device conforms to International Electrotechnical Commission 61672-1:2002. A battery and calibration check was done on the sound-level meter before and after it was used at each site. The sound-level meter was calibrated acoustically in the field before and after every measurement using an external reference (i.e., the sound-level calibrator CR: 514), which was placed over the microphone. The calibrator generated a stabilised sound pressure level of 94 dB (±0.3 dB) at a frequency of 1 kHz.

To avoid reflections from the body of the researcher which could increase the noise level when measurements were being taken from the sound-level meter scale, the instrument was held at arm's length with the microphone pointed at the noise source. The instrument was used to obtain the noise levels from both the churches and the mosques and at the various distances. This was to determine whether the noise produced from these sources was within ambient noise level guidelines of Environmental Protection Agency (EPA), Ghana. Noise emissions need to be observed for at least 30 minutes for the purpose of assessing a duration adjustment if the noise is steady over this period (i.e., there is no observed rising or falling trend in the noise level either audibly or by sound-level meter inspection); a short measurement (e.g., five minutes) should be adequate to represent the 30-minute LAeq [17]. Based on this, the noise readings were taken for 30 minutes for the churches and 15 minutes for the mosques due to the variation in the duration of their activities. For each location, three different noise levels were taken for the night-time and the average noise level was computed. This was done in cognisance of the fact that noise is transient and that noise levels within localities will be varying all the time.

### 2.4. Global Positioning System (GPS)

A Garmin Etrex handheld GPS was used to obtain the coordinates of the churches and mosques and the distances of 50 m, 100 m, 150 m, and 200 m away from the church and mosque within each community.

### 2.5. Calculating Equivalent Noise Exposure Level

This is based on the 8-hour exposure duration for a typical all-night religious service in Ghana. If a resident's (including a worshipper) exposure to noise throughout an all-night service can be characterized by a number of *L*_eq_ measurements for distinct noise activity periods, the measurements can be combined into a full all-night service *L*_eq_ using

(1) Leq, all-night  service=10 Log[(1T)(T1×1010.1L+T2×1020.1L+⋯+Tn×10n0.1L)],

where *T* is the all-night service length, hours, *T*_*n*_ is the duration of the *n*th measurement, hours, and *L*_*n*_ is the *L*_eq_ for the *n*th measurement period, dBA.

For example, if a resident's or worshipper's exposure consists of two distinct exposure periods, 3 hours at an *L*_eq_ of 84 dBA and 5 hours at an *L*_eq_ of 88 dBA, then

(2) Leq8=10 Log[(18){(3×100.1×84)+(5×100.1×88)}]=86.9 dBA=Lex,8.

For an all-night service length greater or less than 8 hours, the *L*_ex,8_ may be calculated using

(3) $$\begin{array}{c} L_{\text{ex},8}=L_{\text{eq},T}+10\;\text{Log}\left(\frac{T}{8}\right), \end{array}$$

where *T* is the religious service duration in hours.

For example, a *L*_eq_ for a 5-hour half-night service would be converted to an *L*_ex,8_ as follows:

(4) $$\begin{array}{c} L_{\text{ex},8}=L_{\text{eq},10}+10\;\text{Log}\left(\frac{5}{8}\right)=L_{\text{eq},10}+1\;\text{dBA}, \end{array}$$

where the night noise exposure is composed of two or more periods of noise exposure of different levels; the following equation may be used to determine if the overall exposure exceeds the allowable noise exposure limits:

(5) $$\begin{array}{c} \left(\frac{C_{1}}{T_{1}}+\frac{C_{2}}{T_{2}}+\cdots +\frac{C_{n}}{T_{n}}\right)\times 100=\%\;\;\text{of}\;\;\text{exposure}\;\;\text{limit}, \end{array}$$

where *C* is the total duration of exposure at a specific noise level and *T* is the total duration of exposure permitted at that level.

The 85 dBA *L*_ex,8_ exposure limit is exceeded when the dose, calculated using the above formula, exceeds 100%. Based on Lutman [18], risk of religious noise-induced hearing loss is minimal if the 8-hour equivalent exposure level is less than 80 dBA. Risk of religious noise-induced hearing loss is significant if the 8-hour equivalent exposure level is greater than 85 dBA.

### 2.6. Data Processing and Analysis

Questionnaires were checked for completeness and internal consistency at the close of each day. Questionnaires were then sorted, numbered, and data-coded before entry into IBM SPSS (version 19) software. GPS noise locations and noise level readings per location were input in ARCGIS 10.1 software.

### 2.7. Spatial Interpolation of Religious Noise Measurements

Since noise is a continuous phenomenon in space, noise levels were converted into a continuous surface using Kriging technique of spatial interpolation. The Kriging interpolation model is known for supplying the best linear unbiased estimate of the level of noise pollution (e.g., ambient noise pollution concentration) at any given location in an area [19]. The principle of Kriging is shown most simply with sets of equations that define the method. Kriging is applied to the estimation of the values of a regionalised variable at a selected location (*Z*_*k*_), based on surrounding existing values (*Z*_*i*_). Each of such locations is assigned a relevant weighting coefficient (*λ*_*i*_), and the calculation of this is the most demanding part of the Kriging algorithm. The value of a regionalised variable can be defined as

(6) $$\begin{array}{c} Z_{i}=Z\left(x_{i}\right), \end{array}$$

where *x*_*i*_ is the value of religious noise at the known location.

Moreover, the value of a regionalised variable estimated by ordinary Kriging based on n points is

(7) $$\begin{array}{c} Z_{k}=\overset{n}{\underset{i=1}{\sum }}\lambda_{i}\cdot Z_{i}, \end{array}$$

where *λ*_*i*_ is the weighting coefficient for a particular location “*i*”; *Z*_*i*_ are known values, the so-called “control points” (hard data); *Z*_*k*_ is the value estimated by Kriging.

These equations represent the system of linear Kriging equations.

Previous equation also can be written as the matrix

(8) $$\begin{array}{c} \left[A\right]\cdot \left[\lambda \right]=\left[B\right]. \end{array}$$

In matrices *A* and *B* the values are expressed as variogram values; that is, these values depend only on the distances and orientations between the control points and not on their values. The third matrix includes weighting coefficients, which are simply estimated from a system with “*n*” equations with “*n*” unknown variables. The Kriging technique (employed in this study) applied some constraints to the matrices, to minimize the error [*σ*_*k*_^2^(*x*)], and this technique gives unbiased estimations. Generally, these factors would describe some external limit (restriction) on the input data, which cannot simply be observed in the measured values. The constraint factor in the ordinary Kriging equations, called the Lagrange multiplicator, was used in this study. If the sum of all weighting coefficients is 1, Kriging expression can be written as

(9) γ(Z1−Z1)×λ1+g(Z1−Z2) ×λ2+⋯+γ(Z1−Zn)×λn+m=γ(Z1−Z),γ(Z2−Z1)×λ1+g(Z2−Z2) ×λ2+⋯+γ(Z2−Zn)×λn+m=γ(Z2−Z),⋮γ(Zn−Z1)×λ1+g(Zn−Z2) ×λ2+⋯+γ(Zn−Zn)×λn+m=γ(Zn−Z)λ1+λ2+⋯+λn+0=1.

If such a system of linear equations is shown as Kriging matrices it can be written as

(10) [γ(Z1−Z1)γ(Z1−Z2)⋯γ(Z1−Zn)1γ(Z2−Z1)γ(Z2−Z2)⋯γ(Z2−Zn)11γ(Zn−Z1)γ(Zn−Z2)⋯γ(Zn−Zn)111⋯10] ×[λ1λ2λnm]=[γ(Z1−Z)γ(Z2−Z)γ(Zn−Z)1].

The number of weighting coefficients and control points can be very large, but contemporary computers can successfully solve numerically demanding tasks. The estimation can be performed simply by calculating the influence of all control points weighted by their associated coefficients according to

(11) $$\begin{array}{c} Z=\lambda_{1}\cdot Z_{1}+\lambda_{2}\cdot Z_{2}+\cdots +\lambda_{n}\cdot Z_{n}. \end{array}$$

The calculation of the estimation variance includes adding the Lagrange coefficient:

(12) σ2=λ1·γ(Z1−Z)+λ2·γ(Z2−Z) +⋯+λn·γ(Zn−Z)+m.

## 3. Results

### 3.1. Night-Time Noise Levels at the Various Noise Sources, 50 m and 100 m Distances

The noise levels recorded at the churches and mosques and the various distances away from the noise sources all exceeded the EPA permissible level of 48 dB(A) for night time (2200–0600 h) expected for residential areas. These are presented in Tables 3 and 4. For night-time (2200–0600 h) noise levels, Grace Assemblies of God Church recorded the lowest value of 71.9 dB and Tribe of Judah Ministries International recorded the highest value of 101.7 dB at the noise source. Pentecost Church at Roman Down and Christ Apostolic Church at Christian Village recorded noise levels of 64.9 dB and 91.4 dB, respectively, at the 50 m distance and 61.6 dB and 83.5 dB for the 100 m distance. Tribe of Judah Ministries International at Taboo Line recorded the highest value of 101.7 dB(A) and Grace Assemblies of God at Market Square recorded the lowest value of 71.9 dB(A). However, at a distance of 50 m from the source, Tribe of Judah Ministries which recorded the highest value at the source recorded 78.5 dB(A) and Grace Assemblies of God which recorded the lowest value at the source also recorded 78 dB(A). The decrease could be attributed to the glass windows used which acted as a form of sound-proof while the increment could be due to other activities aside the church service such as traffic noise. None of the churches or mosques produced noise within the permissible level of 48 dB(A) for night at a distance of 100 m from the church/mosque.

Generally, dose for mosques decreased with increasing distance from the noise source. At the source, the magnitude of dose in decreasing order was as follows: White and Cream Mosque (Zongo Laka) > Green and Cream Mosque (Taifa) > Cream and Green Mosque (Night Market) > Yellow Mosque (Zongo Laka) > White Mosque (Night Market) > White and Green Mosque (Night Market). Residents who are chronically exposed to noise from the first three locations are at significant risk of noise-induced hearing loss whereas residents within the vicinity of White Mosque (Night Market) are at minimum risk. Also, in terms of neighbourhood, Night Market and Zongo Laka areas had the lowest and highest night-time noise exposure, respectively (Table 3).

Regarding churches, all neighbourhoods had at least one location with significant risk of noise-induced hearing loss (Table 4). At the source, the magnitude of dose, for the first ten churches, in decreasing order, was as follows: Tribe of Judah Ministries (Taboo Line) > Musama Church (Market Square) > Christ Apostolic (Roman Down) > Church of Pentecost (Newtown) > Global Evangelical (Newtown) > Church of Pentecost (Christian Village) > Central Assemblies of God (Taifa) > Unique Chapel (Taifa) > Christ Apostolic (Christian Village) > Christ Apostolic (Jericho). In terms of neighbourhood, Market Square and Taboo Line areas had the lowest and highest night-time noise exposure, respectively (Table 4).

### 3.2. Distribution of Night-Time Noise Exposure in the Urban Neighbourhoods

Table 5 shows the classification scheme for night-time noise exposure in the study area. Religious noise exposure up to 84.8 db(A) is regarded as low whereas exposure above 92.6 db(A) is considered extremely high.

**Figure 2:**
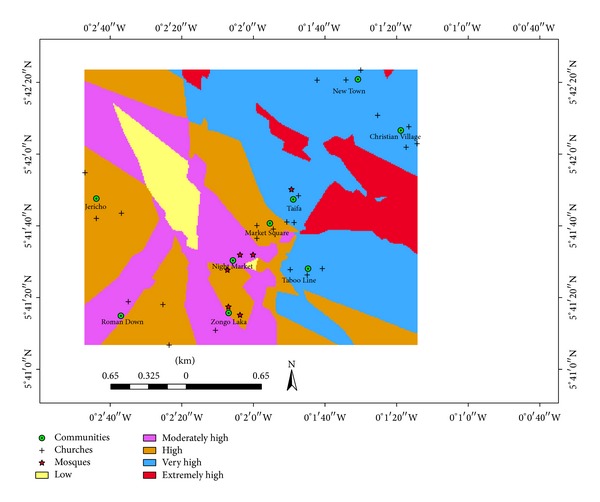
Noise map for* in situ* night-time noise levels in churches and mosques.

As observed in Figure 2, none of the communities was within the low noise exposure class. A major part of Night Market and small portions of Roman Down and Zongo Laka were in the moderately high noise exposure class. Every part of Jericho, major parts of Market Square, Zongo Laka, and Roman Down, and a small portion of Night Market were in the high noise exposure zone. Every part of New Town, Christian Village, Taifa, and Taboo Line and a small portion of Market Square were in the very high noise exposure class. None of the communities was within the extremely high noise exposure class.

## 4. Discussion

This study measured the levels of religious noise produced in the Ashaiman Municipality, a rapidly growing urban environment in Ghana, constructed a night-time noise map indicating the distribution of exposure in residential neighbourhoods of the Ashaiman Municipality, and assessed the risk of religious noise-induced hearing loss to residents in the exposed neighbourhoods. In this municipality there are several worksites for artisanal metal workers in the informal sector. These noise-intensity work environments deliver significant noise to this subset of the people (artisanal metal workers) living in these neighbourhoods. Night-time noise adds to the daytime noise dose. The results showed that all the twenty-three churches and six mosques sampled produced night-time noise levels far exceeding the EPA's guidelines standard of 48 dB(A) for night (2200–0600 h). The noise maps generated also showed that most of the communities were exposed to high noise levels due to religious activities as most of them were within the extremely high, very high, and high noise exposure zones. Just a few communities were within the moderately high zone, but none of the communities was within the low noise exposure zone. The night-time noise levels obtained in this study are comparable to the observations of Armah et al. [9] in the Cape Coast Metropolis. However, the latter study did not control for background noise.

The magnitude of the figure for night-time noise exposure is striking particularly given that excessive night-time noise exposure has the greatest detrimental effect on public health. If the foregoing exposure levels were extrapolated for the entire Accra and Tema areas it quickly becomes obvious that the magnitude of the problem is considerable. The impact that these levels of exposure are having on individual health and the quality of life as well as the economic impact in terms of reduced productively of residents is likely to be considerable also.

Interestingly, there was no statistically significant relationship between neighbourhoods where religious noise exposure was the highest and where noise annoyance was the highest. This finding supports the results Armah et al. [9] obtained in the Cape Coast Metropolis of Ghana. The degree of annoyance produced by noise may vary with the time of day, the unpleasant characteristics of the noise, the duration and intensity of the noise, the meaning associated with it, and the nature of the activity that the noise interrupted [20]. Annoyance may be influenced by a variety of nonacoustical factors including individual sensitivity to noise [21]. These include fear of the noise source, conviction that noise could be reduced by third parties, individual sensitivity, the degree to which an individual feels able to control the noise, and whether or not the noise originated from an important economic activity [20, 22]. Other less direct effects of annoyance are disruption of one's peace of mind, the enjoyment of one's property, and the enjoyment of solitude.

Seldom are populations exposed to a single source of noise pollution. Usually, noise exposure is complex consisting of several simultaneous noise exposures (e.g., religious, air, traffic, etc.). This means that the noise exposure estimates in this study could be far less than the expected composite noise levels. In any case, there is some level of risk of noise-induced hearing loss as shown in this study and the potential effects of exposure cannot be discounted. In fact, the effects of noise exposure depend on several factors, and the absence of a clear dose-effect relationship is certainly due to the complex interactions of these factors, including the noise characteristics, the individual sensitivity, and the context of the explored living environment [2]. However, the amplitude of the subjective complaints about sleep disturbance seems to have been increasing during recent years. This is supported by the growing frequency of noise-related complaints received by the Ghana EPA in the last few years. Unfortunately, only a few epidemiological studies have considered the possible effect of noise exposure (considered globally), together with other environmental factors, on the health of exposed populations. As noted by Fritschi et al. [2], no large-scale epidemiological study focusing on the effect of night-time noise exposure on health has yet been undertaken. Therefore, it is necessary to answer some fundamental questions in order to understand the detrimental effects on health and quality of life in the long-term, for night-time, noise-exposed populations.

Continuous high level exposure can lead to aggression in a hostile, angry, and helpless population. It is often the population with the least income that suffers the most from noise in general. Future epidemiological noise research will need to focus on vulnerable groups, effect modifiers, sensitive hours of the night, coping mechanisms, differences between noise sources, possible confounding with air pollution, differences between objective (noise level) and subjective (noise perception) exposure, and multiple exposures (home, work, and leisure environments).

## 5. Conclusion

This study shows that night-time exposure to religious noise is a growing menace in urban residential areas in Ghana. As expected, noise dose for mosques and churches decreased with increasing distance from the noise source. The magnitude of the figure for night-time noise exposure is striking particularly given that excessive night-time noise exposure has the greatest detrimental effect on public health. All neighbourhoods where churches are situated had at least one location with significant risk of noise-induced hearing loss. However, there was no statistically significant relationship between neighbourhoods where religious noise exposure was the highest and where noise annoyance was the highest. Further research is required to focus on long-term effects of night-time noise exposure of different populations. In this context, the study of specific subgroups can be considered to be “at risk” (e.g., children, elderly people, self-estimated sensitive people, insomniacs, sleep disorder patients, and night worshippers).

## Figures and Tables

**Table 1:** Range for the relationship between nocturnal noise exposure and health effects in the population.

Night-time noise (outside) exposure	Health effects observed in the population
<30 dB(A)	Although individual sensitivities and circumstances differ, it appears that up to this level no substantial biological effects are observed.

30–40 dB(A)	A number of effects are observed to increase: body movements, awakenings, self-reported sleep disturbance, and arousals. The intensity of the effect depends on the nature of the source and the number of events. Vulnerable groups (e.g., children and chronically ill and elderly people) are more susceptible. However, even in the worst cases, the effects seem modest.

40–55 dB(A)	Adverse health effects are observed among the exposed populations. Many people have to adapt their lives to cope with the noise at night. Vulnerable groups are severely affected.

>55 dB(A)	The situation is considered increasingly dangerous for public health. Adverse health effects occur frequently, and a sizable proportion of the population is highly annoyed and sleep-disturbed. There is evidence that the risk of cardiovascular disease increases.

[23].

**Table 2:** Selected communities, churches and mosques, and respondents.

Name of community	Number of respondents		Churches and mosques sampled
	Male	Female	
Taboo Line	9	8	Church of Jesus
			Voice of the Lord Evangelical Church
			Tribe of Judah Ministries International (Int'l)

Market Square	15	14	Apostolic Church, Ghana
			Musama Disco Christo Church
			St. Peter Methodist Church
			Grace Assembly (Assemblies of God (AG))

Taifa	13	13	Central Assemblies of God
			Green and Cream Mosque
			Unique Chapel International (Int'l)

New Town	4	16	Church of Pentecost
			Global Evangelical Church
			Liberty Centre Annex (Assemblies of God (AG))

Christian Village	11	19	Presbyterian Church
			Church of Pentecost
			Christ Apostolic Church
			Evangelistic Fire Ministries International (Int'l)

Zongo Laka	15	5	Grace Chapel International (Int'l)
			White and Green Mosque
			Yellow Mosque

Roman Down	13	6	Church of Pentecost
			St. Augustine Catholic Church
			Christ Apostolic Church

Jericho	7	12	Church of Pentecost
			Church of Christ
			Christ Apostolic Church

Night Market	14	16	White and Green Mosque
			White Mosque (uncompleted)
			Cream and Green Mosque

Total	101	119	

**Table 3:** Night-time noise exposure levels, dose for mosques, and risk of hearing loss in neighbourhoods.

Neighbourhood	Location	Noise level dB(A)∗			Dose (%)			8-hour equivalent exposure level	Risk of noise-induced hearing loss
		Source	50 m	100 m	source	50 m	100 m		
Taifa	Green and Cream Mosque	93.6	76.8	68.6	226.4%	4.7%	0.7%	88.5	Significant risk

Zongo Laka	White and Green Mosque	93.8	85.6	75.2	237.1%	35.9%	3.3%	88.7	Significant risk
	Yellow Mosque	85.6	81.2	72.6	35.9%	13.0%	1.8%	80.5	Some risk

Night Market	White and Green Mosque	84.4	76.2	64.2	27.2%	4.1%	0.3%	79.3	Minimal risk
	White Mosque (uncompleted)	85.4	75.6	64.6	34.3%	3.6%	0.3%	80.3	Some risk
	Cream and Green Mosque	90.2	77.4	64	103.5%	5.4%	0.2%	85.1	Significant risk
	Equivalent noise exposure level	93.2	83.3	73.2	664.30%	66.80%	6.60%		

*Measurement time per location is 15 minutes and total exposure time is 1.5 hours for the mosques.

**Table 4:** Night-time noise exposure levels, dose for churches, and risk of hearing loss in neighbourhoods.

Neighbourhood	Location	Noise dB(A)∗			Dose (%)			8-hour equivalent noise exposure	Risk of noise-induced hearing loss
		Source	50 m	100 m	source	50 m	100 m		
Taboo Line	Church of Jesus	80.1	85.3	72.5	10.1%	33.5%	1.8%	75	Minimal
	Voice of the Lord Evangelical Church	89	81.5	64.8	78.5%	14.0%	0.3%	83.9	Some
	Tribe of Judah Ministries International	101.7	78.5	74.3	1461.7%	7.0%	2.7%	96.6	Significant

Market Square	Apostolic Church, Ghana	86.3	80.5	76.5	42.2%	11.1%	4.4%	81.2	Some
	Musama Disco Christo Church	100.5	81.3	74.7	1108.8%	13.3%	2.9%	95.4	Significant
	St. Peter Methodist Church	83.7	69.7	61.7	23.2%	0.9%	0.1%	78.6	Minimal
	Grace Assembly (Assemblies of God)	71.9	78	74.1	1.5%	6.2%	2.5%	66.8	Minimal

Taifa	Central Assemblies of God	94.8	74.3	73.9	298.4%	2.7%	2.4%	89.7	Significant
	Unique Chapel International	94.7	84.2	67.6	291.6%	26.0%	0.6%	89.6	Significant

Newtown	Church of Pentecost	99.5	83.3	70.5	880.7%	21.1%	1.1%	94.4	Significant
	Global Evangelical Church	98.5	86.9	70.7	699.6%	48.4%	1.2%	93.4	Significant
	Liberty Centre Annex (Assemblies of God)	85.1	74.8	65.3	32.0%	3.0%	0.3%	80	Some

Christian Village	Presbyterian Church	82.5	70.8	63.7	17.6%	1.2%	0.2%	77.4	Minimal
	Church of Pentecost	97.1	83	72.5	506.8%	19.7%	1.8%	92	Significant
	Christ Apostolic Church	94.5	91.4	83.5	278.5%	136.4%	22.1%	89.4	Significant
	Evangelistic Fire Ministries International	93.2	82.5	72.7	206.5%	17.6%	1.8%	88.1	Significant

Zongo Laka	Grace Chapel International	87.3	72.5	62.5	53.1%	1.8%	0.2%	82.2	Some

Roman Down	Church of Pentecost	82.4	64.9	61.6	17.2%	0.3%	0.1%	77.4	Minimal
	St. Augustine Catholic Church	80.9	73.9	64	12.2%	2.4%	0.2%	75.8	Minimal
	Christ Apostolic Church	100	85.1	73.1	988.2%	32.0%	2.0%	94.9	Significant

Jericho	Church of Pentecost	85.7	71.2	63.7	36.7%	1.3%	0.2%	80.6	Some
	Church of Christ	82.3	75.2	64.8	16.8%	24.3%	0.3%	77.2	Minimal
	Christ Apostolic Church	94.3	83.9	74.4	266%	209%	2.70%	89.2	Significant

	**Equivalent noise exposure level**	98.6	88.2	77.7	7327.8%	633.10%	52.10%		

*Measurement time per location is 30 minutes and total exposure time is 11.5 hours for the churches.

**Table 5:** Night-time exposure classes.

Noise range (dB(A))	Exposure class
81.8–84.8	Low
84.8–87.2	Moderately high
87.2–89.9	High
89.9–92.6	Very high
92.6–95.3	Extremely high
